# Hydrocephalus and the neuro-intensivist: CSF hydrodynamics at the bedside

**DOI:** 10.1186/s40635-022-00452-9

**Published:** 2022-05-27

**Authors:** Vasilios Papaioannou, Zofia Czosnyka, Marek Czosnyka

**Affiliations:** 1 Department of Intensive Care Medicine, Alexandroupolis Hospital, Democritus University of Thrace, 68100 Alexandroupolis, Greece; 2 Academic Neurosurgery Unit, Brain Physics Lab, Addenbrooke’s Hospital, P.O. Box 167, CB20QQ Cambridge, UK; 3 Department of Intensive Care Medicine, Alexandroupolis Hospital, Democritus University of Thrace, Polyviou 6-8, 55132 Thessaloniki, Greece

**Keywords:** Hydrocephalus, Cerebrospinal fluid, Shunt, Craniectomy, Cranioplasty, Hydrodynamics

## Abstract

Hydrocephalus (HCP) is far more complicated than a simple disorder of cerebrospinal fluid (CSF) circulation. HCP is a common complication in patients with subarachnoid hemorrhage (SAH) and after craniectomy. Clinical measurement in HCP is mainly related to intracranial pressure (ICP) and cerebral blood flow. The ability to obtain quantitative variables that describe CSF dynamics at the bedside before potential shunting may support clinical intuition with a description of CSF dysfunction and differentiation between normal pressure hydrocephalus and brain atrophy. This review discusses the advanced research on HCP and how CSF is generated, stored and absorbed within the context of a mathematical model developed by Marmarou. Then, we proceed to explain the main quantification analysis of CSF dynamics using infusion techniques for deciding on definitive treatment. We consider that such descriptions of multiple parameters of measurements need to be significantly appreciated by the caring neuro-intensivist, for better understanding of the complex pathophysiology and clinical management and finally, improve of the prognosis of these patients with HCP.

## Take home message

In this review article, we present current and novel theories of CSF circulation and pathophysiology of hydrocephalus, along with results from infusion studies for evaluating CSF dynamics at the bedside.

## Introduction

The origin of the word hydrocephalus (HCP) is Greek and originates from the words ‘hydro’ meaning water, and ‘cephalus’ meaning head. Thus, HCP manifests with excessive cerebrospinal fluid (CSF) accumulation within the brain. Hydrocephalus is not a disease, but a pathologic state with many variations and is supposed to result from a discrepancy between CSF production and absorption. As a result, there is a subsequent accumulation of CSF in the cranial vault with an enlargement of the brain ventricles. In any case, the resulting pressure of CSF against the brain tissue is what causes HCP and for that reason the Hydrocephalus Classification Study Group has defined HCP as ‘an active distension of the ventricular system of the brain resulting from the inadequate passage of cerebrospinal fluid from its point of production within the cerebral ventricles to its point of absorption into the systemic circulation’ [1–3].

Nevertheless, there are conflicting data in the literature regarding mechanistic explanation of ventricular dilatation in patients suffering from subarachnoid hemorrhage (SAH) or after decompressive craniectomy [3]. Different authors have questioned the classical model of CSF circulation, asking for a broader definition of HCP that focuses on cranial fluid dynamics [4], whereas implementation of novel MRI techniques have changed the way we understand the physiology of CSF flow within the central nervous system (CNS) [5]. In this respect, Linninger and colleagues [6] advocated for a ‘holistic model of the physics of the central nervous system that incorporates the interaction between blood flow, cerebral vasculature expansion, soft tissue stresses and CSF dynamics including production, flow and reabsorption’.

In addition, apart from third ventriculostomy, implantation of a shunt is a standard way of managing HCP. Since shunt is a mechanistic treatment that affects patient’s pressure–volume compensation, the hydrodynamics of each patient’s compensatory reserve should be tested before a shunt is implanted. Testing CSF dynamics at the bedside, even during patient’s stay in the Intensive Care Unit (ICU), is an invasive method but may help with the decision regarding CSF diversion or cranioplasty after decompressive craniectomy [7].

In this review, we present data from recent studies regarding novel theories of CSF circulation and pathophysiology of HCP, in patients suffering from subarachnoid hemorrhage. In addition, we provide selected forms of physiological measurements in HCP using infusion studies that were performed in the Neurocritical Care Unit at Addenbrooke’s hospital in Cambridge, UK. We think that a neuro-intensivist should be familiarized with such methods for better understanding pathophysiology of HCP, as well as for deciding proper management for each patient according to multiple forms of measurements that constitute the basics of CSF dynamics.

### CSF: the third circulation

Generally, CSF dynamics depends on interaction between four components: production, flow, absorption and pulsations.

#### Classic hypothesis of CSF hydrodynamics

CSF is secreted by the epithelial cells of the choroid plexuses. These cells like those of other secretory epithelia are polarized in a way that the properties of their apical membrane are different from those of the basolateral membrane. Apical membrane is made up of numerous microvilli and the basolateral membrane has many infoldings, whereas both membranes have a greatly expanded area. As a result, the total area available for transport is similar to that of the blood–brain barrier. About 500 ml of CSF is produced daily and the choroid plexuses is responsible for 60–70% of CSF production. The remaining 30–40% comes from the interstitial fluid exuded from the pia from vessels. CSF physiology is based on the active formation of CSF, passive absorption and unidirectional flow of CSF from the site of formation to the place of absorption. CSF circulation is referred to as the third circulation (the other two are blood and lymph) [8, 9]. CSF flows from the lateral ventricles through the foramen of Monro into the third ventricle and then on into the fourth ventricle via the cerebral aqueduct. Subsequently, CSF empties out of the fourth ventricle via the midline foramen of Magendie and the lateral foramina of Luschka into the subarachnoid space (SAS), which comprises a network of interconnected CSF cisterns located around the basal aspect of the brain. Once in the SAS, the CSF flows over the cortical convexity and skull base until its final reabsorption at the arachnoid granulations (AGs) into the superior sagittal sinus. CSF provides physical protection of the brain and spinal cord in cases of trauma, reducing the active weight of the nervous structures, according to the Pascal law [7]. Secondly, all pressure gradients are cancelled out by free circulation of CSF. Furthermore, CSF may allow clearance of different metabolites and toxins from the brain. In any case, ‘its most significant task is to allow for an even distribution of pressure throughout the intracranial vault, reducing any pressure gradients and preventing brain shifts or herniation’ [7]. Weed and Dandy [9, 10] were the first who described CSF circulation, which was subsequently analyzed mathematically by Davson [11].

**Fig. 1:**
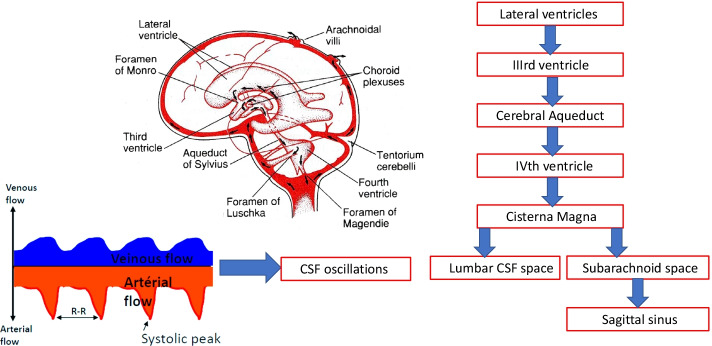
CSF circulation. Overview of the ventricular system. CSF is produced and flows from the choroid plexuses in the lateral ventricles into the third and then fourth ventricle via the foramina of Monro and the cerebral aqueduct, respectively. From the IVth ventricle, CSF empties into the cisterns of the skull base through the foramen of Magendie and foramina of Luschka and subsequently into the lumbar CSF space and the subarachnoid space at the sagittal sinus. Cardiac contraction induces an arterial distension during systole and a subsequent recoiling during diastole. A portion of this energy is transferred to the brain in the form of brain pulsation and to the CSF in the form of CSF pulsation. This dissipation of arterial blood flow energy by the CSF pulsation energy provides for the maintenance of low intracranial pressure (ICP) according to the Windkessel effect on CSF flow

CSF circulates in a to-and-fro movement with a caudal-directed net flow through the brain ventricles to SAS, exchanging various substances. In addition, CSF circulates not only in a constant way with a rate equivalent to its production, but also in pulsations, as has been observed in the cerebral aqueduct, as well as in the cervical region of SAS [4, 5]. Such pulsations have been attributed to the Windkessel effect of CSF drainage. This effect is the energy transfer due to arterial blood flow energy formed by cardiac contraction, which is transferred into the cranial cavity as arterial blood flow, brain pulsations and CSF pulsation [5]. Thus, for a half of the cardiac cycle, CSF flows down into the spinal subarachnoid space (the 5th ventricle) and for the other half, upward into cranial compartments. As a result, each pulse sets a pressure gradient throughout the CNS, which tends to force CSF out of the cerebral ventricles. In conclusion, CSF is actively produced from the choroid plexuses (CSF pumps), then it circulates slowly towards the SAS and is subsequently passively absorbed into the venous sinuses by the arachnoid villi (AGs) (Fig. 1) [3].

#### New insights into CSF hydrodynamics

However, novel findings from different experimental studies have questioned the accuracy of Dandy’s theory [10] since the secretion of CSF seems to be a pressure-dependent process and decreases as ICP is increased [3]. In addition, it seems that CSF is not only absorbed into the venous sinuses but rather inside the ventricles, as well as the choroid plexuses and the lymphatic system. Furthermore, it has been found that CSF has also an extra-choroid origin and is formed except in the ventricles, within the SAS [3, 8]. Finally, it has been shown that there is no net formation of CSF in isolated brain ventricles but rather than permanent CSF changes happen within the surrounding tissue, depending on fluid osmolarity [3]. According to different theories, since water constitutes 99% of CSF volume, it is apparent that water demonstrates the dynamics of CSF, indicating that CSF does not actually circulate according to classic hypothesis, but rather continuously produced and reabsorbed throughout the whole CNS [3, 12].

In this respect and based on the theory of Klarica and colleagues [3, 13], the interstitial fluid (ISF) and CSF water constitute a functional unity and are regulated by both osmotic and hydrostatic pressure in CNS microvessels. Thus, a continuous turnover of ISF-CSF is created by the filtration of water across the capillary walls under hydrostatic pressure and the reabsorption of water follows from the interstitium into the venous capillaries by osmotic counter-pressure. In this respect and since the surface of choroid plexuses is about 5.000 times smaller than the surface of cerebral capillaries, it is assumed that both formation and absorption of CSF takes place at the cerebral capillaries [3].

According to the poroelastic model [6], resorption of the ISF–CSF brain fluid occurs at multiple sites along perivenous Virchow–Robin spaces, perineural sheaths of cranial and spinal nerves, meningeal lymphatics along dural sinuses, arachnoid villi and interosseous connections between meninges and the skull. The term ‘glymphatic pathway’ describes the exchange of CSF–interstitial brain fluid via the Virchow–Robin perivascular spaces and subsequent drainage from the CNS by a plethora of anatomic sites, including recently discovered cranial lymphatics [14]. The vascular pulsations of the brain and CSF hydrostatic pressure are considered significant factors in the movement of CSF through the SAS and into the brain parenchyma through the glymphatic system. The presence of endothelial tight junctions across the cerebral vasculature inhibits enter of CSF flow that travels along Virchow–Robin spaces into cerebral blood vessels or the brain parenchyma [4].

### Pathophysiology of post-SAH hydrocephalus

Post-SAH ventricular dilatation may have a wide range of aetiological factors: starting from neuronal loss due to possible secondary ischemic insults, to obstruction of CSF circulation resulting in hydrocephalus [7]. Hydrocephalus (HCP) is a serious and common complication in the clinical course of SAH. A wide range between 6 and 67% of incidence of HCP in SAH patients has been observed in different studies, whereas the most recent data report an incidence of 20–30% [15]. Post-SAH HCP can be classified as: (1) acute, occurring with 72 h post-ictus in about one fifth of patients; (2) subacute occurring over 2–3 weeks and (3) chronic, occurring more than one month after the initial injury, resulting in permanent HCP often treated via CSF diversion [16, 17]. Regardless of the occurring period, HCP impairs patients’ neurologic function and results in significant neurological disability, coma and even death. Risk factors that predispose individuals to HCP include poor Hunt–Hess grade, intraventricular hemorrhage, history of hypertension and rupture of aneurysms in the posterior circulation that can lead to obstruction of the fourth ventricle [15, 16].

HCP in general is considered as a pathological state rather than a simple excessive accumulation of CSF within the ventricles and spinal canal [3]. This pathological condition is the result of different pathophysiological processes, such as inflammation, bleeding, trauma, increased ICP and increased CSF osmolarity, which sometimes overlap between each other.

#### Obstructive HCP

Based on the classical theory of CSF hydrodynamics, HCP may develop due to an obstruction of different circulating pathways (obstructive HCP), a reduction in its absorption from the AGs (communicating HCP) or an overproduction of CSF [10, 11]. In any case, such theory is based on the classical experiment of Dandy in dogs in 1919 [10], which showed that HCP is always caused by an obstruction of different CSF pathways, due to either a space-occupying lesion or an inflammatory insult. The dilatation of the ventricular system is therefore the result of the CSF pathway obstruction with a parallel CSF accumulation, produced from CSF pumps in front of the obstruction. A pressure gradient across the cerebral mantle, which is called transmantle pressure and is the difference between intraventricular pressure and pressure inside the SAS of the cerebral convexity, is responsible as a driving force for such ventricular dilatation [18]. In that case and since CSF is formed exclusively from the choroid plexus, its surgical removal was suggested as the most appropriate treatment [3, 10]. For many years choroid plexectomy was the most popular form of HCP treatment. Nevertheless, despite removal of the source of CSF production, ventricles remained enlarged, giving rise to an open question that has raised a lot of debate in the literature: since active CSF formation does not exist why would the aqueduct obstruction lead to ventricular enlargement?

Different experimental studies support the hypothesis that aqueductal narrowing or even closure occurs as a result of HCP instead of being its cause [19]. It seems that as hydrocephalic state progresses, axial herniation and compression of the midbrain lead to aqueduct stenosis as a secondary phenomenon [3, 18, 19]. Moreover, Klarica and colleagues [13] demonstrated that in cases of complete acute aqueductal blockage, CSF pressure in ventricles was identical to control conditions and that brain ventricles did not dilate during 3 h of obstruction.

#### Communicating HCP

Communicating HCP occurs very frequently and it is believed to represent an impairment of CSF absorption at the level of AGs, associated with increased resistance to CSF outflow. In such cases, all parts of the ventricular system are dilated, whereas the aqueduct of Sylvius remains patent and posteriorly expanded. In addition, alterations in the lymphatic pathway have recently been proposed as another pathophysiological mechanism of CSF absorption. Such pathway constitutes a route through the cribriform plate into the external lymphatic system located in the nasal submucosa [4, 14]. However, such theories do not answer the question of how does obstruction of CSF absorption cause ventricular dilatation. It is postulated that a transmantle pressure gradient precedes ventricular enlargement but it is not clear how this pressure gradient is originated. In this case, such gradient associated with impaired CSF absorption at the level of AGs should favor expansion of SAS but not the ventricles. Moreover, CSF pressure should rise equally in all CSF spaces within the cranium [3].

A true transmantle pressure gradient can develop only in cases of total blockade of the CSF pathway between the ventricles and SAS with a simultaneous increase in CSF volume in front of the blockade. Thus, an early post-SAH HCP might be due to an abrupt blockade of the ventricular system at the level of aqueduct, probably associated with a clot formation after bleeding into the CSF. Subsequently, CSF volume continues to increase due to increased osmolarity of CSF that is associated with blood presence within CSF compartments. Increasing osmolarity leads to water influx from surrounding tissue and to an increase in CSF volume. Finally, such effects may augment ICP and give rise to a transmantle pressure gradient that induces ventricular dilatation [3].

Different experiments using gated spin-echo MRI sequences and cine phase-contrast MRI to measure CSF hydrodynamics have shed more light into the pathophysiology of ventricular enlargement in cases of communicating HCP [20]. In healthy adults, it seems that during each heart beat where a particular volume of blood is injected into the cranium, CSF flushes (venting) from the intracranial SAS into the cervical spinal SAS, regulating instantaneously ICP. In addition, the brain pulsates caudally during systole, squeezing the SAS and causing CSF flow and mixing, like an ‘expanding and retracting piston’ [4, 20]. On the contrary, ventricular CSF flow represents less than 6% of the intracranial blood variation and occurs at almost 20–25% of the cardiac cycle duration [5]. Thus, in normal conditions, the ventricular system plays a minor role in the dampening of the brain systolic expansion, while the ‘CSF mobile compliance’ of the intracranial cavity depends mainly on the SAS [21]. However, in cases of post-SAH communicating HCP, a decreased intracranial compliance induces an increase of the systolic pressure transmission into the brain parenchyma. Such increase can distend the brain towards the skull and simultaneously compress the periventricular region against the ventricles, giving rise to a ventricular enlargement and narrowing of the SAS. In this case, restricted CSF oscillations in the intracranial SAS compartment increase CSF oscillations in the ventricles to maintain cervical CSF oscillations, then balance vascular expansion and prevent a large increase in ICP during the cardiac cycle [3, 22, 23]. In conclusion, it seems that development of communicating HCP could be the result of the redistribution of CSF pulsation in the cranium due to the dissipation of arterial pulsation into the SAS and the flow of the stronger arterial pulsations to the choroid plexus and both capillary and venous circulation. This pulse pressure gradient that is created between the ventricles and the SAS causes ventricular dilatation at the expense of the SAS.

Another potential mechanism associated with ventricular enlargement includes CNS venous insufficiency [24, 25]. Any increase in CNS volume causes compression of the veins that might result in increased venous pressure, a reduction in the blood flow out of the CNS and finally, a reduction of CSF absorption back into the blood circulation. Consequently, an increase in CNS volume could lead to CSF accumulation and development of HCP [25]. Apart from a reduction in venous compliance in the territory drained by the superior sagittal sinus, bridging veins are compressed within the ventricles during each cardiac pulsation. Thus, CSF intracranial compartment distends its spaces during blood injection into the choroid plexuses through compression of veins, such as bridging veins, within its confines, until all possible venous collapse has occurred. Such phenomenon has been described as a moving resistance by volume venting into the jugular venous system at each pulse, giving rise to a damped CSF wave [26]. However, in cases of reduced venous venting with minimal potential for venous collapse, due to reduced compliance, inflammatory wall induration and hardening, moving resistance and damping cannot occur [26, 27]. In such cases, the movement of CSF is rapidly stopped by the increased resistance (reduced venous venting) and high pulse pressure (amplitude) results, associated with increased CSF pulsatility within the ventricles.

### Post-craniectomy HCP

Hydrocephalus after decompressive craniectomy for refractory intracranial hypertension is a common finding in patients suffering from traumatic brain injury (TBI) and SAH and raises significant diagnostic challenges to the carrying physician in the Neurocritical Care setting [28]. Its pathophysiology differs from that of post-SAH hydrocephalus and its understanding might ameliorate accurate decision-making regarding prompt therapy and recovery. Post-craniectomy HCP has also been described as a syndrome of the Trephined (SoT), characterized most commonly by unexplained neurological dysfunction. Different studies and meta-analyses have shown that there is a correlation between severity of initial TBI or SAH, as well as the presence of parafalcine (interhemispheric) subdural hygroma [29, 30]. Although CSF diversion through ventricular shunting can improve ventriculomegaly, hygromas in general do not respond well to shunting alone and require cranioplasty [4]. In a recent review of complications after decompressive craniectomy [31], frequency of HCP was 14.8% after TBI, 21.1% after SAH and cerebral hemorrhage and 25.5% after ischemic stroke. Delayed abnormal cranial fluid collections that can impair normal CSF flow or absorption are common after decompressive craniectomy and when they cause clinical worsening, they have also been called external hydrocephalus [4]. Finally, the development of SoT with HCP is more likely when cranioplasty is delayed more than 3–6 months.

According to recent experimental data, HCP after decompressive craniectomy is usually related to: (1) reduced internal brain expansion due to the large compliant cranial defect; (2) reduced CSF pulsatile flow along major cerebral arteries and the Virchow–Robin spaces; (3) reduced clearance of the interstitial fluid by the glymphatic system and (4) redistribution of CSF from the SAS to the subdural and subgaleal compartments and the ventricles [4].

Regarding CSF pulsatility, decompressive craniectomy creates a large low-resistance defect in the rigid cranial vault. In this case the Monro–Kellie doctrine that the sum of all contents within the skull is a fixed volume is not valid. As a result, normal brain pulsatile inward expansion is dampened and ventricular squeezing during each pulsation is diminished, giving rise to a flattened ICP waveform [4, 32]. In addition, ipsilateral cerebral blood flow has been found to be reduced [33]. It is assumed that external barometric pressure on the scalp is transmitted to the cerebral vasculature, causing decreased blood flow to the area of the defect [34]. Both cerebral blood flow, CSF and brain pulsatility can be improved after cranioplasty, which can also be an adequate treatment to avoid permanent shunt implantation. Moreover, CSF diversion through shunting is not indicated in cases of significant brain atrophy due to necrotic cell death [4, 7]. Such extensive necrosis of brain tissue can also be associated with post-craniectomy HCP, and for that reason, performance of CSF hydrodynamic tests at the bedside can guide clinicians towards the appropriate treatment [35].

### CSF hydrodynamics at the bedside: model of CSF circulation and infusion studies in post-SAH hydrocephalus

The mathematical model of CSF pressure–volume compensation has been advanced by professor Marmarou who was the first who integrated all components-CSF production, circulation, absorption and storage-in one elegant theoretical structure expressed as an electric circuit [7, 36]. This model provides a theoretical basis for the differential diagnosis of hydrocephalus (HCP). Consequently, HCP is now characterized using parameters from this model such as resistance to CSF outflow, elasticity and pressure–volume index (PVI) [37]. Marmarou also proposed a mathematical description of the linear relationship between pulse amplitude (AMP) of intracranial pressure (ICP) and mean values of ICP, as well as ICP’s vascular component [37]. It seems that in patients with traumatic brain injury (TBI), only 30% of cases of elevated ICP can be explained by changes in CSF circulation [38].

Under normal conditions, production of CSF is balanced by its storage and reabsorption into the sagittal sinus (SG) according to Eq. 1:

1 $${\text{CSF production }} = {\text{ CSF storage }} + {\text{ CSF reabsorption}},$$

CSF production is almost constant and is related to ICP according to Davson equation [11]:

2 $${\text{ICP }}\left( {\text{CSF pressure}} \right) = R{\text{ outflow}} \times {\text{ CSF production }} + \, P_{{{\text{ss}}}} ,$$

where *R* is the resistance to CSF outflow (units: mmHg × ml^−1^ × min^−1^) and *P*_ss_ is the pressure at the level of sagittal sinus.

Reabsorption is proportional to the gradient between CSF pressure (*P*) and pressure in SG (*P*_SS_):

3 $${\text{Reabsorption of CSF }} = \, P - P_{{{\text{SS}}}} /R,$$

*P*_SS_ is determined by central venous pressure.

Storage of CSF is proportional to CSF compliance C (units: mmHg × ml^−1^).

4 $${\text{Storage}} = C* \, \left( {{\text{dp}}/{\text{dt}}} \right).$$

The compliance *C* of CSF space is inversely proportional to the gradient of CSF pressure *P* and a reference pressure *P*_0_ [39]:

5 $$C \, = \, 1/\left[ {E*\left( {P - P_{0} } \right)} \right].$$

The coefficient *E* is termed cerebral elasticity (or elastance coefficient) (units: ml^−1^). Elevated *E* (> 0.18 ml^−1^) signifies a poor pressure–volume compensation [40]. In general, Eq. 5 expresses the most important law of CSF dynamics: when CSF pressure increases, the compliance *C* of the brain decreases, respectively.

Combination of Eqs. 1 with 2 and 5 gives the final Eq. 6 [36]:

6 $$\left\{ {1/\left[ {E*\left( {P - P_{0} } \right)} \right]} \right\}*{\text{dp}}/{\text{dt }} + \left( {P - P_{b} } \right)/R \, = \, I\left( t \right),$$

where *I*(*t*) is the rate of external volume addition and *P*_*b*_ is the baseline pressure.

Equation 6 can be solved for various types of external volume administrations *I*(*t*). Nevertheless, the most common in clinical practice is a bolus infusion of CSF (volume Δ*V*), giving rise to a pressure *P*(*t*) that describes the shape of the relationship between the effective volume increase Δ*V* and the CSF pressure, called the pressure–volume curve). The bolus injection can be used for calculation of the so-called pressure–volume index (PVI), defined as the volume added externally to produce a tenfold increase in CSF pressure [7, 36]:

7 $${\text{PVI }} = \, \Delta V/\log \, \left( {P_{p} - P_{0} /P_{b} - P_{0} } \right) \, = \, 1/0.434*E,$$

where *P*_*p*_ is the peak pressure recorded after addition of the volume Δ*V*. PVI is theoretically proportional to the inverse of brain elastance coefficient E and whenever pressure–volume compensatory reserve is insufficient, PVI is lower than 13 ml. On the contrary, a PVI higher than 26 ml signifies and over-compliant brain. Such values are valid in cases of slow infusion. However, if the bolus test is used, norms of PVI are higher (the threshold equivalent to 13 is around 25 ml) [7].

Practically, the bolus injection involves a fast, 2–10 s addition of 5–10 ml of Hartman solution into the CSF space. This test gives rise a value of PVI from the fast phase of rising of ICP. Resistance to CSF outflow can be calculated from the time constant of decay of the raised ICP back to baseline values.

Regarding AMP, if we presume that the rise in blood volume after each heart contraction is equivalent to a rapid bolus addition of CSF fluid at the baseline pressure *P*_*b*_, the pulse amplitude can be estimated according to Eq. 8 [39]:

8 $${\text{AMP }} = \, P_{p} - P_{b} = \, \left( {P_{b} - P_{0} } \right) \times \left( {e^{E\Delta V} - 1} \right).$$

In almost all cases, AMP is increased upon CSF increase by an external volume addition. The gradient of the regression line between AMP and ICP is proportional to *E* [7, 39]. However, such model suffers from specific limitations. Thus, it cannot capture dynamic interactions between rising ICP and cerebral blood volume.

The aim of all kinds of CSF volume–pressure studies is to measure *R* along with other compensatory parameters in cases of HCP. Almost all researchers agree that during hydrocephalus, *R* is increased and CSF drainage is disturbed. The limit of normal resistance is reported to range between 13 and 18 mmHg × ml^−1^ × min^−1^.

**Fig. 2:**
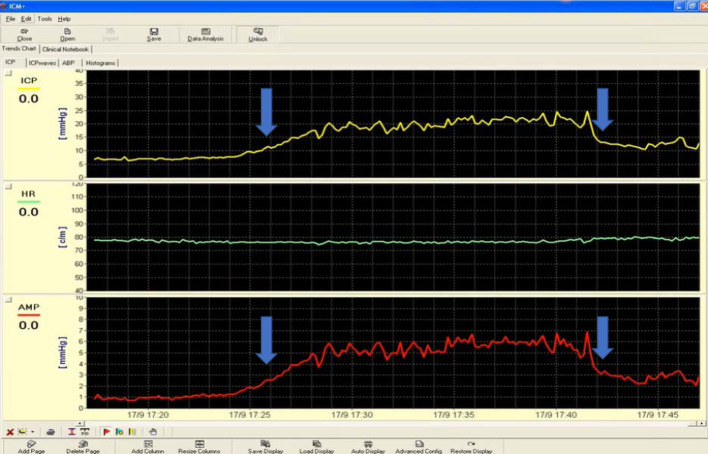
Example of normal CSF circulation during a constant rate infusion study. Infusion test of a patient with normal CSF circulation. Opening (before starting the infusion) intracranial pressure (ICP) is around 6 mmHg and estimated *R* = 8 mmHg × min/ml. The blue arrows indicate the beginning and the end of the infusion test. HR refers to heart rate and AMP to the amplitude of ICP (pulse pressure). A parallel increase in AMP with ICP is noticed

The computerized infusion test developed by Czosnyka [7, 41] is a modification of the traditional constant rate infusion that was firstly described by Katzman [42], whose application is limited to communicating HCP. It lacks the limitations of the bolus injection that requires a significant amount of injected liquid in order to create a sudden and observable transient increase in intracranial pressure, whereas gradual infusion suggests adding fluid in a controlled manner. The method requires a constant fluid infusion into CSF compartment (Fig. 2). A lumbar infusion or an intraventricular infusion into a subcutaneously positioned reservoir, connected to an intraventricular catheter is needed for such measurements. Lumbar infusion has significant limitations in unstable ICU patients but is less invasive than intraventricular. In the second case, two hypodermic needles are used: one for the pressure measurement and the second for the infusion. Although the precise measurement of the final plateau pressure is not always possible, for reasons of safety and in order to avoid excessive elevation of intracranial pressure, a safety limit of 40 mmHg is used. Even in cases of increased intracranial pressure when the infusion is terminated prematurely without reaching the end-plateau, the computer can calculate mean pressure and pulse amplitude and estimates *R* to CSF outflow as the difference between the value of the plateau pressure during infusion and the resting pressure divided by the infusion rate. The algorithm utilizes advanced time-series analysis for volume-pressure curve retrieval, least-mean-square model fitting and an examination between AMP and ICP. Moreover, the elastance coefficient *E* or the PVI, cerebrospinal compliance, CSF formation rate and different indices of pressure–volume compensatory reserve are also estimated. Regarding infections rate, its incidence is less than 1% according to data from internal audit in Addenbrooke’s hospital.

**Fig. 3:**
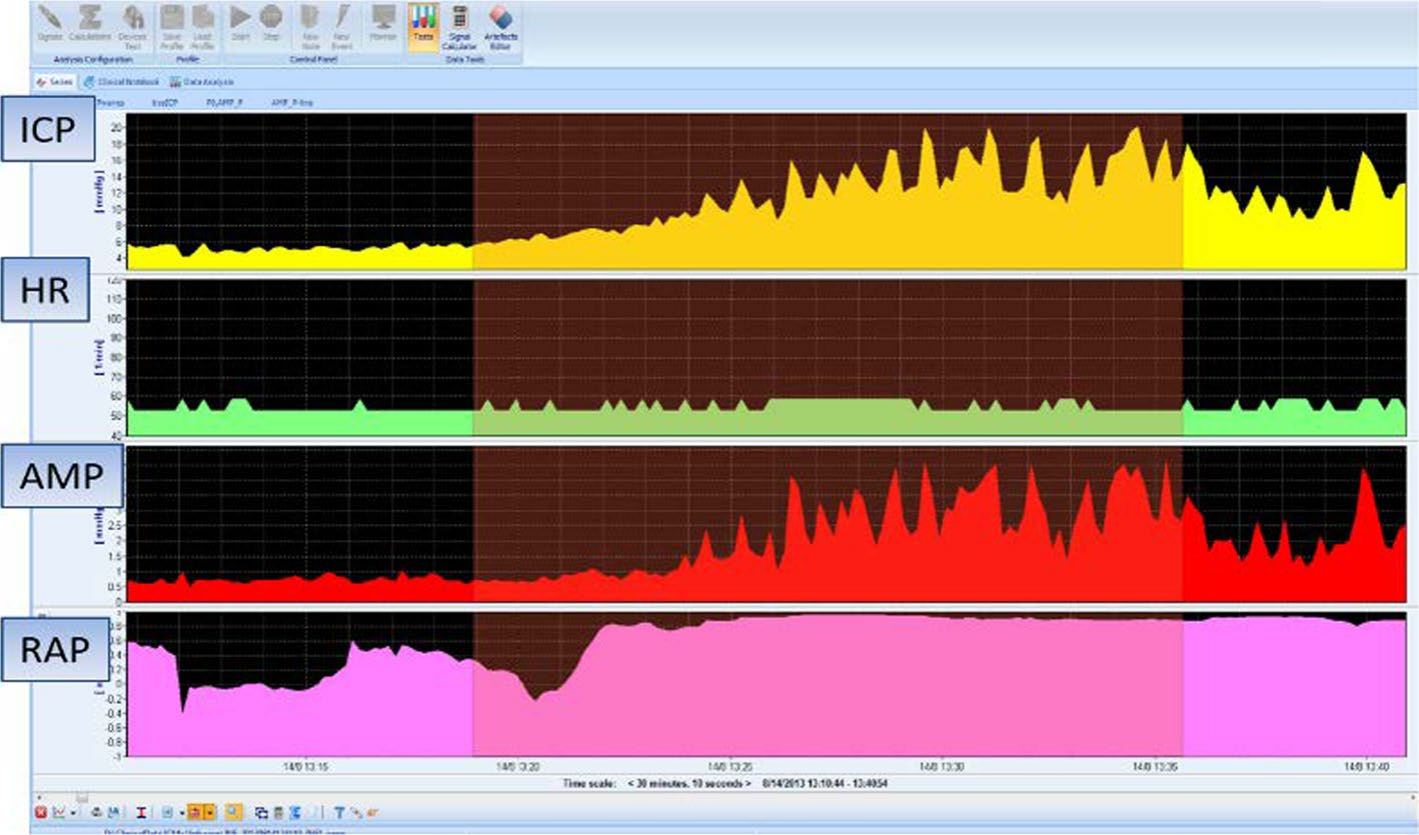
Example of a constant rate infusion test in a post-SAH patient with HCP. HCP with normal baseline ICP. The dark red area depicts infusion period. Although baseline ICP was normal (4.75 mmHg), it rises until 36.5 mmHg until the end of the infusion study (plateau pressure), whereas the *R* to CSF outflow (Rcsf) is increased (18.59 mmHg × ml^−1^ × min^−1^). There are also a lot of spikes in both ICP and AMP (amplitude) tracings reflecting strong vasogenic B waves. Changes in AMP are well correlated with changes in ICP. The RAP index that is the correlation coefficient between the pulse amplitude AMP and the mean value of ICP is close to + 1, signifying poor compensatory reserve. This is a case of post-SAH communicating HCP

In this respect, the software can calculate the so-called RAP index that is the correlation coefficient between the pulse amplitude AMP and the mean value of ICP, derived by linear correlation between 40 consecutive, time averaged data points of AMP and mean ICP, acquired within a 6 s wide time window [7]. RAP describes the relationship between ICP and changes in intracerebral volume (pressure–volume curve). A RAP index close to 0 indicates a lack of coupling between changes in AMP and mean ICP, denoting a good pressure–volume compensatory reserve. However, when this curve starts to increase exponentially, AMP co-varies with mean ICP and consequently RAP rises to + 1. This indicates low compensatory reserve (Fig. 3).

**Fig. 4:**
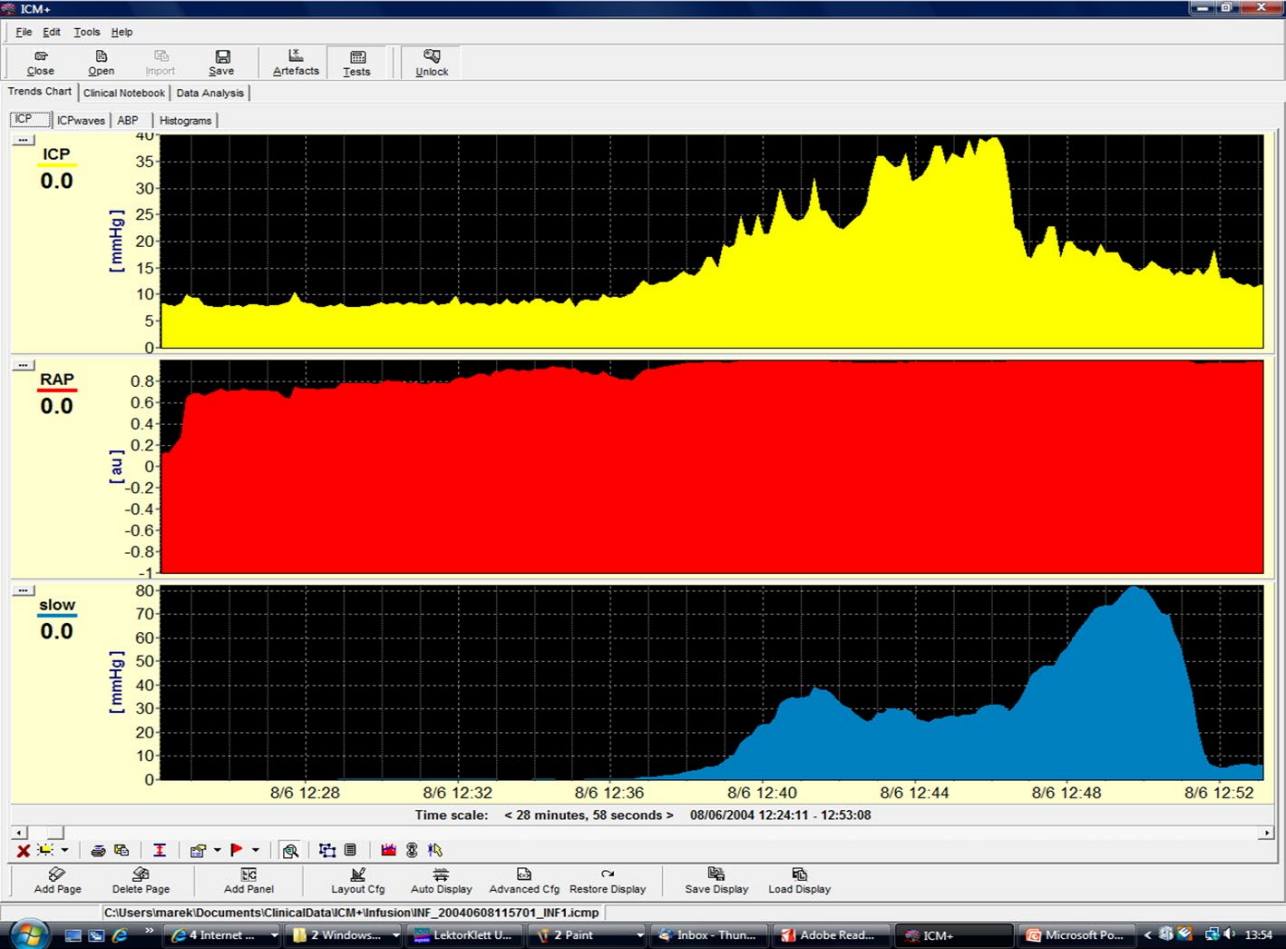
Example of a constant rate infusion test in post-SAH HCP with B (slow) waves. The same patient as in Fig. 3 where a RAP index close to + 1 is associated with an increased power of B (slow) waves during infusion test. A fast Fourier transformation (FFT) was performed to evaluate the energy (power) of B waves within the ICP signal

**Fig. 5:**
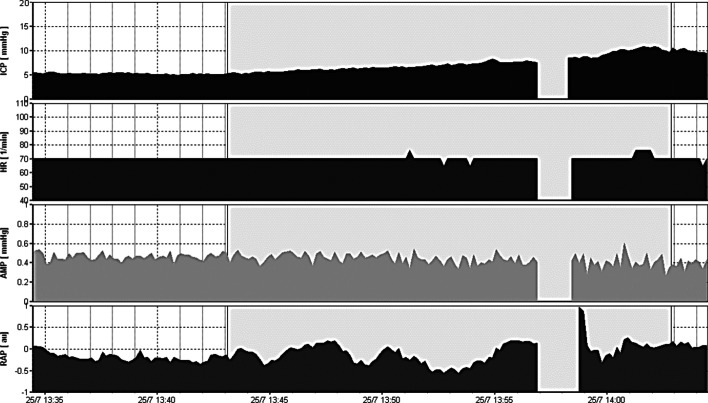
Example of a constant rate infusion test in a post-SAH patient with brain atrophy. ICP during the infusion study (gray area) remains low, increasing from 5.24 mmHg to 10.36 mmHg (plateau values). There is no increase in either RAP index or AMP (amplitude), whereas there is a lack of B waves during infusion (absence of significant spikes in ICP and AMP signals). Elasticity and resistance *R* to CSF outflow are 0.04 ml^−1^ and 3, 4 mmHg × ml^−1^ × min^−1^, respectively. Such low values indicate brain atrophy rather than hydrocephalus

Parameters describing vascular effects and pressure–volume compensation can also be estimated during the infusion study. According to classical standards, when so-called B waves are present in more than 80% of ICP monitoring time, shunting is recommended in cases of HCP [43]. B waves are slow waves of ICP associated with fluctuations in the tone of cerebral vessels. They have a period from 20 s to 2 min and are almost universally present. However, their presence with an amplitude greater than 1 mmHg for a duration longer than 15 min signifies a pathologic level of B wave [44]. B waves are correlated with fluctuations of cerebral blood flow velocity [45] and their absence reflect brain atrophy rather than increased *R* to CSF outflow (Figs. 4 and 5). According to the theory of increased CSF pulsatility within the ventricles as a cause of ventricular enlargement, the increased presence of B waves during an infusion study might be associated with increased transmission of arterial blood pulsations within the brain [7].

**Fig. 6:**
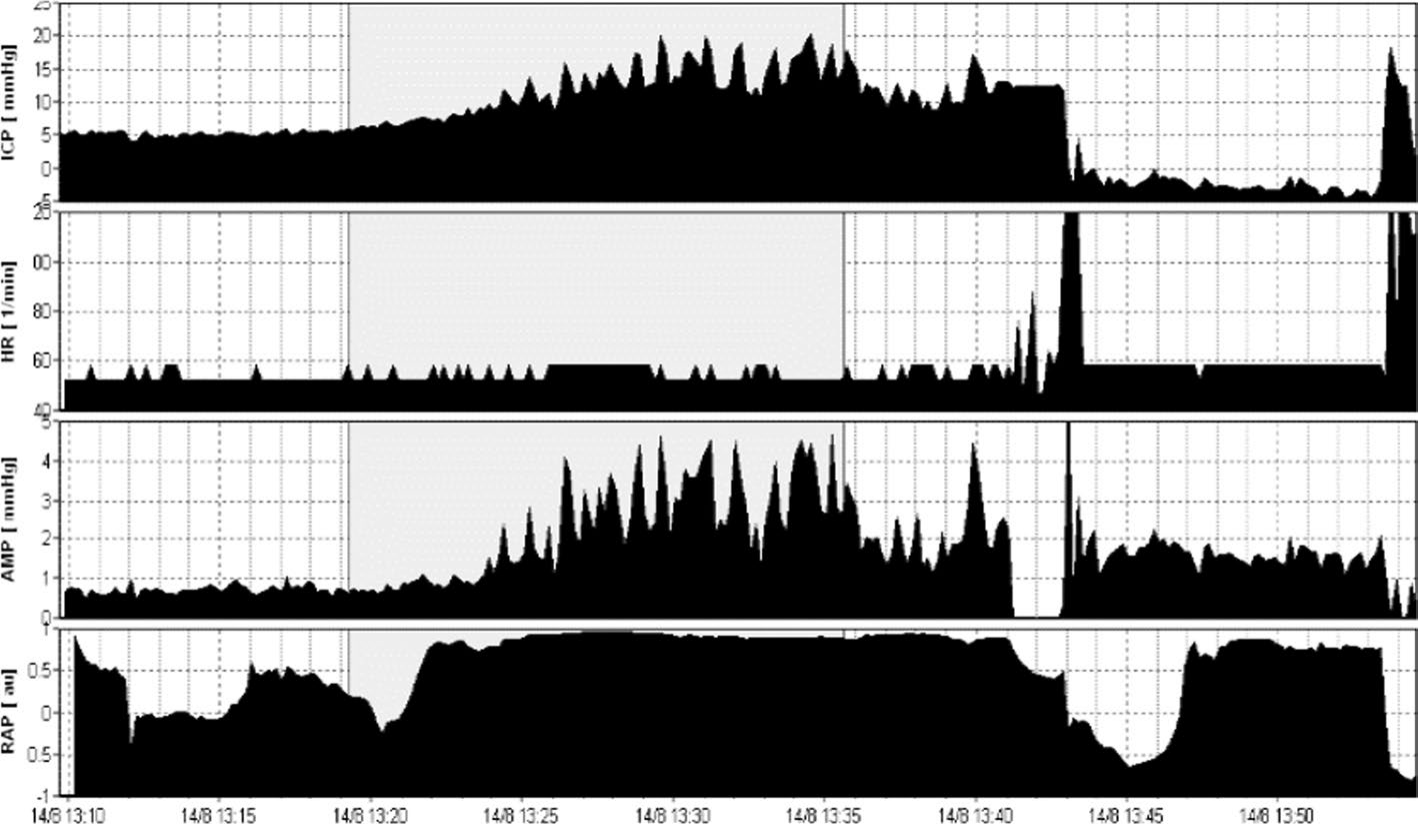
Example of a constant rate infusion test in a patient with craniectomy and brain atrophy. In this post-craniectomy, post-SAH patient, baseline and plateau pressures during infusion test (gray area) are normal (5.33 and 15.45 mmHg, respectively). Resistance *R* to CSF outflow is low (7.61 mmHg × ml^−1^ × min^−1^) but there are some B waves in the tracings of both ICP and AMP (pulse amplitude). Nevertheless, their energy and duration are lower in relation with cases of disturbed CSF circulation. This patient does not need any shunt. RAP index is close to + 1 during infusion test, signifying poor pressure–volume reserve

Patients suffering predominately from brain atrophy have normal CSF circulation. Typically, baseline ICP, *R* to CSF outflow and AMP are low (*R* < 10 mmHg × ml^−1^ × min^−1^). The RAP index is less than 0.6, reflecting low elasticity of the atrophic brain (*E* < 0.2 ml^−1^). Furthermore, B waves are rather limited during infusion studies (Fig. 6). Finally, ICP increases smoothly during infusion and decreases in a similar function following infusion [7, 46]. On the contrary, obstructive HCP can be safely assessed using ventricular infusion, demonstrating a high resistance to CSF outflow (*R* > 13 mmHg × ml^−1^ × min^−1^). The elasticity is high (*E* > 0.20 ml^−1^) and RAP index is elevated more than 0.6 with an AMP higher than 4 mmHg, indicating poor compensatory reserve. Acute communicating HCP during post-SAH presents with a similar pattern of parameters, with frequent vasogenic waves [7, 46, 47].

### Infusion studies in post-craniectomy hydrocephalus

Post-craniectomy ventricular dilatation may have a wide range of etiological factors: from neuronal death due to head trauma and subsequent secondary ischemic insults to obstruction of CSF circulation resulting in HCP [35]. It is important to differentiate post-craniectomy HCP and brain atrophy before considering placement of a shunt. Such decisions can be facilitated by performing an infusion test.

**Fig. 7:**
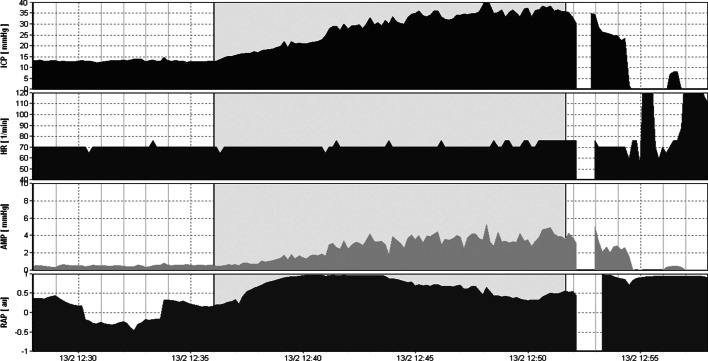
Example of a constant rate infusion test in a post-SAH and post-cranioplasty patient. The same patient as in Fig. 6 upon cranioplasty 6 months later during an infusion study. The patient exhibits a high plateau ICP during infusion (35 mmHg), a high RAP index close to + 1 with elevated elasticity (0.25 ml^−1^), indicating poor compensatory reserve and increased resistance *R* to CSF outflow (15 mmHg × ml^−1^ × min^−1^). This case illustrates disturbed CSF circulation and the need for a limited period without a bone flap, something that only an infusion study might confirm

In addition, CSF circulation may change significantly after a cranioplasty, resulting from a previous decompressive craniectomy for refractory ICP elevation. In general, the *R* to CSF outflow after craniectomy decreases twofold and brain compliance (as evaluated with PVI) increases substantially [35]. According to Shapiro et al. [48], time constant of CSF circulation (*R* to CSF outflow × compliance of CSF space) has a tendency to remain constant. In this respect, a mechanistic increase in compliance after craniectomy tends to be followed by a decrease in *R*. This process might be reversed after cranioplasty, meaning a decrease in PVI might be followed by an increase in resistance to CSF outflow. According to another theory, a large craniectomy may facilitate irreversible ventricular enlargement [35]. Thus, after cranioplasty, the expanded ventricle could obstruct the lumen of the cortical subarachnoid space through the cerebral mantle and increase subsequently R to CSF outflow (Figs. 6 and 7).

### External lumbar drainage and tap test

External lumbar drainage (ELD) test is based on the idea of simulating the operation of the shunt by withdrawing a certain amount of CSF for long time durations, usually 48–72 h, and can be used at an inpatient setting [49]. Tap test is a method for partial replacement of ELD and involves the removal of about 40–50 ml of CSF within 15–30 min. The drainage rate depends on patient’s condition, such as age and brain atrophy. Its use is currently implemented in an outpatient clinic following an infusion test and aims at comparing different symptoms, such as gait impairment or cognitive function before and after CSF removal, therefore a good patient communication is necessary. For that reason, it is not recommended in sedated patients in the ICU.

## Conclusions

In this review, we tried to present data supporting the theory of HCP origin based on altered brain pulsatility and CSF dynamics in both post-SAH HCP and after decompressive craniectomy. In addition, it seems that physiological monitoring through infusion studies in patients developing ventricular enlargement after SAH or decompressive craniectomy can be useful in deciding if the patient is suffering from HCP, needing permanent treatment through shunting or cranioplasty, or if he is exhibiting significant brain atrophy due to secondary ischemic insults, which is resistant to CSF diversion through shunt. In any case, implementation of CSF hydrodynamic studies at the bedside may improve understanding CSF circulation, helping the carrying physician to apply more efficient and beneficial treatment for patients.

## Data Availability

Not applicable.
